# The Presence of Non‐HLA Antibody With DSA Is Associated With Moderate to Severe T Cell‐Mediated Rejection in Liver Transplant Recipients

**DOI:** 10.1111/ctr.70480

**Published:** 2026-02-15

**Authors:** Qingyong Xu, Nigar A. Khurram, Carol Bentlejewski, Anthony J. Demetris, Adriana Zeevi

**Affiliations:** ^1^ Division of Transplant Pathology, Department of Pathology University of Pittsburgh Pittsburgh Pennsylvania USA

**Keywords:** antibody, DSA, HLA, liver transplantation, Non‐HLA

## Abstract

**Background:**

Antibodies to donor HLA (DSA) and non‐HLA antigens are associated with detrimental outcomes in kidney, heart, and lung transplants. Such data are scarce in liver transplants (LTx). We aim to study the roles of DSA and non‐HLA antibodies in T‐cell‐mediated rejection (TCMR) of LTx.

**Methods:**

Allograft biopsies (*n* = 103) from adult LTx recipients were studied. Biopsy‐paired serums were retrospectively tested for anti‐angiotensin II type 1 receptor (AT1R) and the Luminex panel of 60 non‐HLA Antibodies.

**Results:**

TCMR was detected in 59 of 103 (57.3%) biopsies. Twenty‐six biopsies were categorized as moderate‐severe (MS‐TCMR), 77 as negative‐mild (NM‐TCMR). DSA was positive in 95/103 (92.2%) cases and wasn't associated with MS‐TCMR. Anti‐AT1R antibodies were elevated in serum paired with MS‐TCMR vs. NM‐TCMR (18.8[15.2–40.0] vs. 13.0[10.0–21.5] U/ml, *p* < 0.01). Positive anti‐AT1R antibodies were associated with a higher incidence of MS‐TCMR (HR = 12.4[1.5–101.6], *p* = 0.02). A panel of 18 non‐HLA Abs was significantly associated with MS‐TCMR. The number of panel‐18 non‐HLA antibodies was higher with MS‐TCMR vs. NM‐TCMR (3[2–5] vs. 1[0–1], *p* < 0.001). The incidence of MS‐TCMR was higher in cases with panel‐18 non‐HLA antibodies ≥3 vs. <3 (HR = 19.6[6.0–64.8], *p* < 0.001). The frequency of MS‐TCMR was the highest when DSA, anti‐AT1R, and panel‐18 non‐HLA antibodies were all present.

**Conclusions:**

Non‐HLA antibodies to AT1R or the Luminex panel are associated with MS‐TCMR in LTx biopsies. The incidence of MS‐TCMR is higher when non‐HLA antibodies are present concomitantly with DSA, indicating an additive effect. Further studies are warranted to investigate the utility of routinely monitoring DSA and non‐HLA antibodies in LTx recipients.

**Abbreviations**
ARHGDIB: Rho GDP dissociation inhibitor beta
AT1R: Angiotensin II type 1 receptor
AUC: Area under the curve
DSA: Donor‐specific antibody
GSTT1: Glutathione S‐transferase theta‐1
HLA: Human Leukocyte Antigen
HR: Hazard Ratio
IQR: Interquartile Range
LG3: Laminin‐like globular domain of Perlecan
MFI: Mean fluorescence intensity
MS‐MVI: Moderate to severe microvascular inflammation
MS‐TCMR: Moderate to severe T‐cell mediated rejection
MVI: Microvascular inflammation
NM‐TCMR: Negative to mild T‐cell mediated rejection
NM‐MVI: Negative to mild microvascular inflammation
ROC: Receiver operating characteristic
SNRPB: Small nuclear ribonucleoprotein polypeptides B
STAT6: Signal Transducer and Activator of Transcription 6
TCMR: T‐cell Mediated Rejection

## 1 Introduction

The human leukocyte antigen (HLA) is the primary barrier to successful allogeneic transplantation. Donor‐specific HLA antibodies (DSA) are associated with the rejection of kidney, heart, liver, and lung transplants [1, 2]. However, the detrimental impact of DSA on liver transplant outcomes is generally accepted, but inconsistently reported in the literature [3, 4, 5, 6, 7]. One explanation is that the liver is rather resistant to alloimmune responses; thus, prolonged exposure to much stronger DSA is often necessary for the injury to manifest in liver function tests. Additionally, immunity to non‐HLA targets may act synergistically with alloimmunity in damaging the liver allografts [8], similar to reports in the kidney [9], heart [10], and lung [11, 12] allografts.

Previously, we reported that pre‐formed non‐HLA antibodies to the AT1R (Angiotensin II type 1 receptor), LG3 (laminin‐like globular domain of Perlecan), and DSA were strongly associated with poor graft survival in adult liver retransplantation [13, 14]. However, posttransplant circulating DSA/non‐HLA antibodies were not monitored, nor was the histology of liver regraft biopsies. In pediatric transplant recipients with liver allograft dysfunction, positive DSA and anti‐AT1R antibodies identify patients at high risk for rejection and graft loss [15]. Non‐HLA antibodies, such as anti‐AT1R, may trigger complement deposition and endothelial activation in the microvasculature, thereby inducing a distinctive phenotype of antibody‐mediated rejection [8, 9, 16].

Using multiplex bead assays that test multiple non‐HLA antibodies in a single assay, several groups have reported that the presence of an increased number of non‐HLA antibodies was associated with a deleterious outcome in the kidney [17], heart [10], and lung [11] transplant recipients. In a cohort of pediatric liver transplant recipients, we identified a panel of 3 non‐HLA antibodies—SNRPB (small nuclear ribonucleoprotein polypeptides B), GSTT1 (Glutathione S‐transferase theta‐1), and Actin—that were associated with T cell‐mediated rejection (TCMR) synergistically with DSA [18].

Here, in a cohort of adult liver transplants, we examined the role of DSA and non‐HLA antibodies, such as anti‐AT1R and Luminex panels, in TCMR.

## 2 Materials and Methods

The inclusion criteria are: 1) Adult liver transplant recipients at UPMC (2014–2023); 2) liver biopsies with a DSA testing serum collected within a month of biopsy date; and 3) available sera for retrospective testing of non‐HLA antibodies. Rejections were retrospectively graded by a single pathologist, based on the 1997 Banff criteria [19], blinded from the results of DSA and non‐HLA antibody tests. The Banff schema of TCMR is based on cumulative scores for portal inflammation, bile duct damage, and subendothelial inflammation [19]. This study was approved by the Institutional Review Board (IRB #19010210).

Banked posttransplant sera were retrospectively tested with a Luminex‐based solid‐phase assay for 60 non‐HLA antibodies (LIFECODES Non‐HLA Antibody, Immucor, Norcross, GA, Lot#3013217). Mean fluorescence intensity (MFI) was used for data analysis. Vendor‐provided cutoffs were used to determine the positive beads. To select a panel of relevant Luminex non‐HLA antibodies significantly associated with moderate‐severe TCMR (MS‐TCMR), Chi‐square tests (*p* < 0.05) were performed to identify specific non‐HLA antibodies.

Anti‐AT1R antibodies were tested retrospectively with an ELISA kit (One Lambda, West Hills, CA). Positivity for anti‐AT1R was categorized based on a vendor‐provided cut‐off: < 10 U/mL as negative, 10–17 U/mL as “at risk”, and > 17 U/mL as positive. The concentrations of anti‐AT1R antibodies were maximally recorded at the upper detection limit of 40 U/ml.

As part of routine patient care, anti‐HLA antibodies were tested prospectively using the Luminex Single Antigen Beads assay, with a mean fluorescence intensity (MFI) greater than 1000 as the positive threshold (One Lambda, West Hills, CA). The donor reactivity for anti‐HLA DSA was determined based on complete donor low‐intermediate level HLA molecular typing for HLA‐A*, B*, C*, DRB1/3/4/5*, DQA1/B1*, and DPA1/B1*. For DSA with MFI > 8000, a C1q single antigen bead test was performed to detect complement‐fixing antibodies. Only the DSA information in the biopsy‐paired serum was used in the analysis for its associations with rejections and non‐HLA antibodies.

The variables were compared using the Mann–Whitney test for quantitative variables and the Chi‐Square test or Fisher's exact test for qualitative variables, respectively. Binary logistic regression was used to calculate the hazard ratio (HR) and the *p*‐value. Statistical analyses were performed with IBM SPSS version 29.0.2.0.

## 3 Results

### 3.1 Demographics

One hundred three for‐cause biopsies with serum pairs from 78 patients were included. Sixty‐two patients (79.5%) had only one biopsy, nine patients (11.5%) had two biopsies, six patients (7.7%) had three biopsies, and one patient had five biopsies included in the study (Table S1). Biopsies were performed at a median of 366 days (Interquartile range [IQR]: 171–732) posttransplant. Serums were collected at a mean of 2 ± 9 days after biopsy. The demographics for study patients are summarized in Table S2. They are mostly male (60.8%), white (86.1%), with an average age of 53.2 ± 13.9 years. Most of the studied liver transplants are the first graft (91.1%). The common primary diseases are alcoholic cirrhosis (29.1%), NASH (Non‐alcoholic steatohepatitis, 22.8 %), malignancy (19.0%), PSC (Primary Sclerosing Cholangitis, 12.7%), and viral infections (8.9%).

### 3.2 Elevated Non‐HLA Antibodies in Serum Are Associated With Moderate to Severe TCMR

**FIGURE 1:**
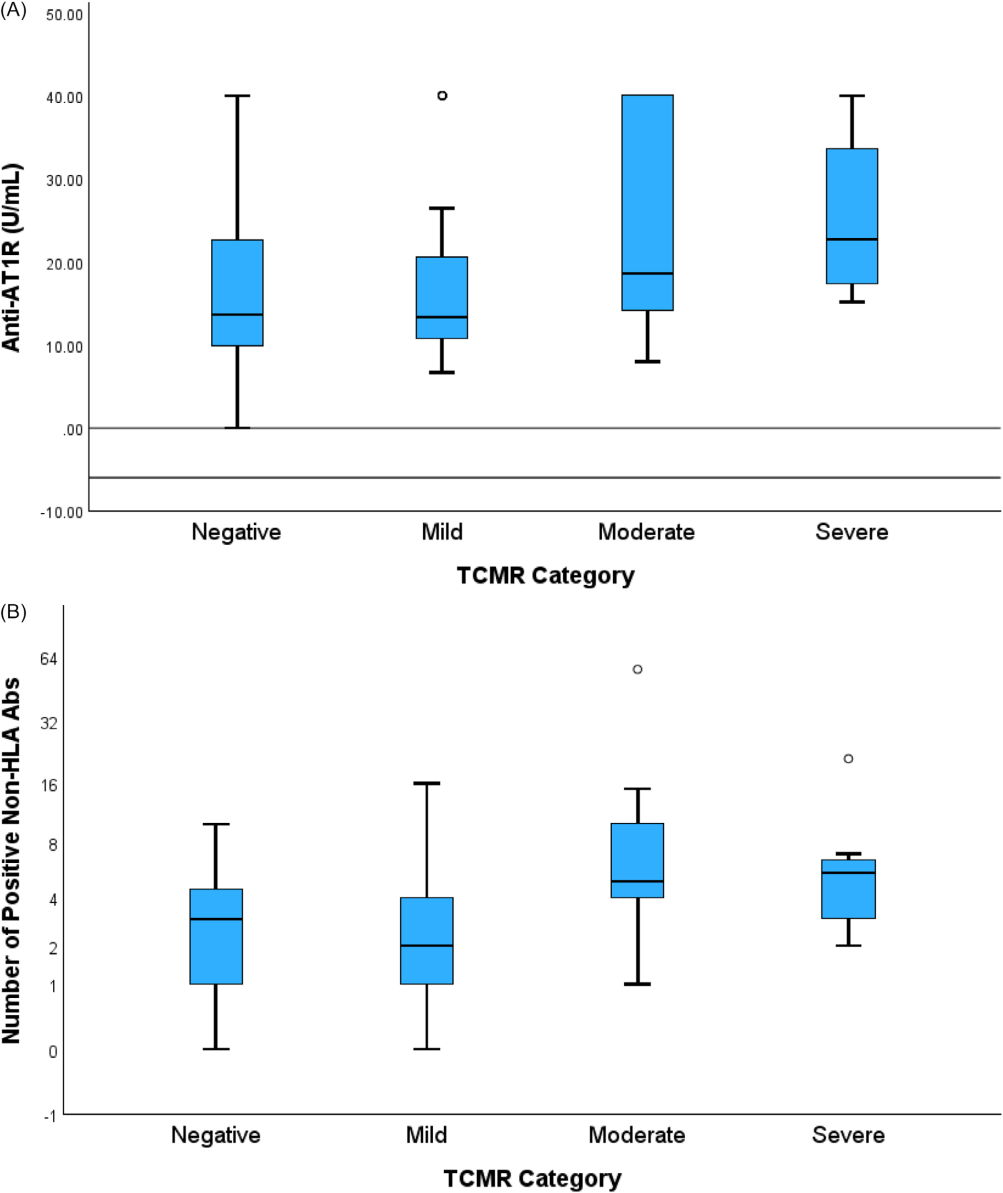
(A) Anti‐AT1R (Angiotensin II type 1 receptor) antibodies were elevated in moderate‐severe than in negative‐mild TCMR (T cell‐mediated rejection). In the Kruskal–Wallis test, overall *p* = 0.03; *p* < 0.05 for negative vs. moderate, negative vs. severe, mild vs. severe; *p* = 0.067 for mild vs. moderate; *p* > 0.10 for other pairwise comparisons. (B) Serum samples paired with moderate‐severe TCMR (T‐cell mediated rejection) had an increased number of Luminex non‐HLA antibodies compared to those paired with negative‐mild TCMR. In the Kruskal–Wallis test, overall *p* = 0.002; *p <* 0.05 for negative vs. moderate, negative vs. severe, mild vs moderate, mild vs. severe; *p* > 0.05 for other pairwise comparisons.

TCMR was found in 59/103 biopsies (57.3%), with 33 cases classified as mild, 18 cases as moderate, and 8 cases with severe TCMR; the remaining 44 cases were negative for TCMR. Significantly higher concentration of anti‐AT1R antibodies was found in serum paired with moderate/severe TCMR vs. negative TCMR, and in those with severe vs. mild TCMR (*p* < 0.05, Figure 1A). There was an insignificant trend toward higher anti‐AT1R levels in those with moderate versus mild TCMR (*p* = 0.067, Figure 1A). If any positive non‐HLA antibodies were detected with the 60‐antibody Luminex panel and numerated as the overall burden, the numbers were significantly higher in serum paired with moderate/ severe TCMR compared to those with negative/mild TCMR (*p* < 0.05, Figure 1B). Conversely, neither the anti‐AT1R levels nor the number of any positive non‐HLA panel antibodies are significantly different between mild and negative TCMR, or severe and moderate TCMR (Figure 1). Thus, from this point forward throughout the paper, biopsies were categorized into two groups: Negative‐Mild (NM‐TCMR, *n* = 77) and moderate‐severe TCMR (MS‐TCMR, *n* = 26).

**FIGURE 2:**
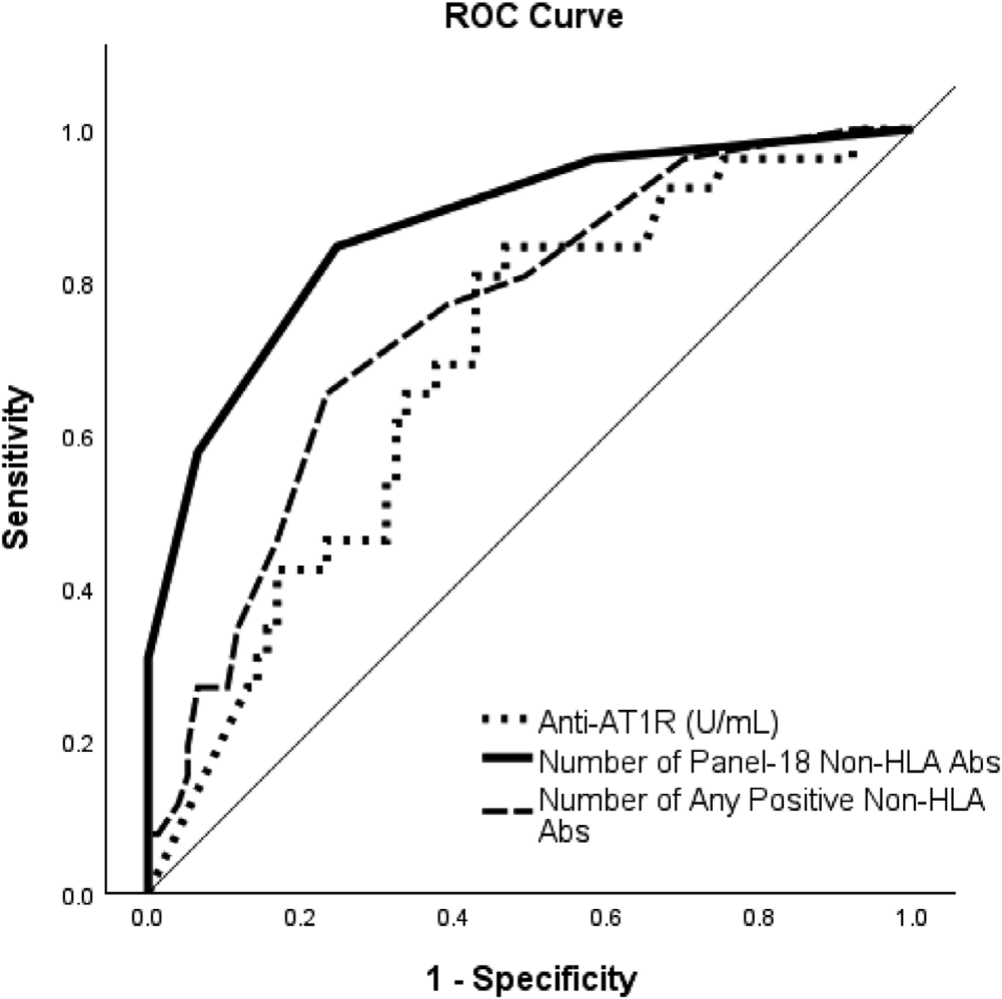
ROC analysis for variables associated with moderate‐severe T cell‐mediated rejection. AT1R, Angiotensin II type 1 receptor; AUC, area under the curve; ROC, receiver operating characteristic.

Anti‐AT1R antibodies were elevated in patients with MS‐TCMR as compared to NM‐TCMR (Median [IQR]: 18.8 [15.2–40.0] vs. 13.0 [10.0–21.5] U/ml, *p* < 0.01, Table 2 and Figure S1A). The concentration of anti‐AT1R antibody distinguishes MS‐TCMR from NM‐TCMR in receiver operating characteristic (ROC) analysis (Area under the curve [AUC] = 0.69 ± 0.06, *p* = 0.001, Figure 2). The increasing concentration of anti‐AT1R antibodies was associated with a higher incidence of MS‐TCMR (HR = 1.05 [1.01–1.09] per incremental U/ml antibody, *p* = 0.01, Table 2). Patients with positive anti‐AT1R antibodies (>17 U/ml) had a significantly higher incidence of MS‐TCMR (HR = 12.4 [1.5–101.6], *p* = 0.02, Table 2) compared with those with negative anti‐AT1R antibodies (<10 U/ml). Patients with anti‐AT1R at risk (10–17 U/ml) exhibited a trend toward an increased incidence of MS‐TCMR (HR = 4.75 [0.55–40.98], *p* = 0.156).

Eighteen non‐HLA antibodies were significantly associated with a higher incidence of MS‐TCMR as compared to NM‐TCMR (*p* < 0.05). These non‐HLA antibodies are summarized in Table 1. Except for anti‐α‐Enolase (*n* = 38), anti‐GSTT1 (*n* = 17), and anti‐Rho GDP dissociation inhibitor beta (ARHGDIB, *n* = 19) antibodies, most other non‐HLA antibodies have a low incidence (n ≤ 13) in the cohort of 103 cases, thus having a very low positive predictive value. Hence, a panel of 18 non‐HLA antibodies was used to measure the overall cumulative burden of non‐HLA antibodies, similar to what was reported in assessing non‐HLA antibodies in the kidney [17], heart [10], and lung [11] transplant recipients.

**TABLE 1:** A panel of 18 non‐HLA antibodies is significantly associated with moderate‐severe TCMR.

Antigen	Antigen Detail (positive cutoff in MFI)	NM‐TCMR (*N* = 77)	MS‐TCMR (*N* = 26)	*p*
AGRIN	Agrin (1718)	0	3	0.002
ARHGDIB	Rho GDP dissociation inhibitor beta (1298)	10	9	0.014
ATP5B	ATP synthase, beta polypeptide (867)	5	6	0.018
CD40	CD40, TNF receptor superfamily member 5 (430)	0	3	0.002
CXCR9	C‐X‐C Motif Chemokine 9 (532)	1	3	0.019
DEXI	Dexamethasone‐induced transcript (836)	1	5	<0.001
ENO1	Alpha‐enolase (2413)	22	16	0.003
FLRT2	Leucine‐rich repeat transmembrane protein 2 (457)	1	5	<0.001
GSTT1	Glutathione S‐Transferase theta‐1 (587)	9	8	0.023
HARS	Histidyl‐tRNA synthetase, Jo‐1 (1910)	1	3	0.019
LGALS3	Lectin, galactoside‐binding, soluble, 3 (455)	2	4	0.016
NCL	Nucleolin (2142)	2	6	<0.001
P2RY11	Purinergic receptor P2Y, G‐protein coupled, 11 (765)	0	4	<0.001
PLA2R1	Phospholipase A2 receptor 1, 180 kDa (477)	0	2	0.014
PTPRO	Receptor‐type Tyrosine‐protein Phosphatase U (573)	0	4	<0.001
SHC3	SHC Adaptor Protein 3 (1205)	5	7	0.005
SNRPN	Small Nuclear Ribonucleoprotein Polypeptide N (Smith antigen core) (1634)	4	5	0.028
STAT6	Signal Transducer and Activator of Transcription 6 (840)	6	7	0.011

*Note:* *p* values were calculated with the Chi‐squared test.

Abbreviations: MFI, mean fluorescence intensity; MS‐TCMR, moderate‐severe T cell‐mediated rejection. NM‐TCMR, negative‐mild T cell‐mediated rejection.

A higher number of panel‐18 non‐HLA antibodies was found in patients with MS‐TCMR than those with NM‐TCMR (median [IQR]: 3 [2–, 5] vs. 1 [0–1], *p* < 0.001, Figure S1B and Table 2). ROC analysis (Figure 2) demonstrated that the number of panel‐18 non‐HLA antibodies (AUC = 0.87 ± 0.04, *p* < 0.001) significantly distinguishes MS‐TCMR from NM‐TCMR more effectively than unselected, any positive non‐HLA antibodies with Luminex panel‐60 (AUC = 0.75 ± 0.05, *p* < 0.001). An increased number of panel‐18 non‐HLA antibodies was associated with a higher incidence of MS‐TCMR (HR = 3.83 [2.10–6.99] per incremental non‐HLA Ab, *p* < 0.001, Table 2), indicating a cumulative effect of non‐HLA antibodies on MS‐TCMR. For cases with panel‐18 non‐HLA antibodies of 3 or more, the incidence of MS‐TCMR was higher than for those with fewer antibodies (HR = 19.6 [6.0–64.8], *p* < 0.001, Table 2).

**TABLE 2:** Antibodies associated with moderate‐severe TCMR.

	NM‐TCMR (*n* = 77)	MS‐TCMR (*n* = 26)	HR (95% CI)	*P*
Anti‐AT1R (U/ml), Median (IQR)	13.4 (10.0–21.1)	18.8 (15.1–40.0)	1.05 (1.01–1.09)	0.012
Anti‐AT1R positivity, n				
Negative (<10U/ml)	19	1	1	
At‐risk (>10, < 17 U/ml)	32	8	4.75 (0.55–40.98)	0.156
Positive (> 17U/ml)	26	17	12.42 (1.52–101.63)	0.019
Number of positive Panel‐18 Non‐HLA Antibodies, Median (IQR)	1 (0–1)	3 (2–5)	3.83 (2.10–6.99)	< 0.001
Number of Panel‐18 Non‐HLA Antibodies, n				
≤ 2	72	11	1	
≥ 3	5	15	19.64 (5.95–64.84)	< 0.001
DSA (MFI > 5K), Panel‐18 non‐HLA Antibodies ≥ 3, Anti‐AT1R (> 17U/ml)				< 0.001*
All negative	21	0		
Single‐positive	36	8		
Double‐positive	19	12		
Triple‐positive	1	6		

*Note:* HR (hazard ratio), 95% CI (Confidence Interval), and *p*‐values were calculated using binomial logistic regression unless otherwise specified. *p‐value was calculated with the Chi‐squared test.

Abbreviations: AT1R, Angiotensin II type 1 receptor; DSA, donor‐specific antibody, only DSA in the biopsy‐paired serum was analyzed; IQR, Interquartile Range; NM‐TCMR, negative‐mild T cell‐mediated rejection; MS‐TCMR, moderate‐severe T cell‐mediated rejection.

### 3.3 DSA Is Not Associated With Moderate to Severe TCMR

When using an MFI greater than 1000 as the positive cutoff, DSA was positive in most biopsy‐paired serum samples from this cohort (95/103, 92.2%). Most DSA‐positive sera had DSAs directed against HLA class II only (*n* = 67), followed by those directed against both class I and class II (*n* = 21), and those directed against class I only (*n* = 7). Out of 95 DSA‐positive cases, 10 sera had pre‐existing DSAs (10.5%), 70 sera had *de novo* DSAs (73.7%), and 15 sera had both pre‐existing and *de novo* DSAs (15.8%). The presence of positive DSAs in the biopsy‐paired serums, at thresholds of 1000 MFI, 10000 MFI, or positive with the C1q assay, was not associated with MS‐TCMR (Table S3). There was an insignificant trend towards high incidence for MS‐TCMR with a 5000 MFI cutoff (HR = 1.28 [0.80–2.06], *p* = 0.30, Table S3)

### 3.4 Additive Effect of DSA and Non‐HLA Antibodies on TCMR

Published literature demonstrates that the DSA with MFI > 5000, either performed [20] or *de novo* [21], are associated with allograft dysfunction and reduced survival, sometimes synergistically with anti‐AT1R antibodies [8]. Considering the high incidence of positive DSA in this cohort, we investigated whether there is an additive effect of MS‐TCMR when DSA and non‐HLA antibodies are present concomitantly (Table 2). An increased frequency of MS‐TCMR was observed in patients with all three analytes: positive DSA with MFI > 5000, the presence of more than 3 non‐HLA panel‐18 antibodies, and positive anti‐AT1R antibodies (>17 U/ml) (Table 2). The majority, 6 out of 7 (85.7%) of triple‐positive cases were MS‐TCMR. In contrast, none of the 21 triple‐negative cases had MS‐TCMR (0/21, 0%). The double‐positive cases (12/31, 38.7%) have a higher incidence of NM‐TCMR than the single‐positive ones (8/44, 18.2%).

## 4 Discussion

The increased incidence of DSA and multiple non‐HLA antibodies, including anti‐AT1R and a non‐HLA Luminex panel, in cases with MS‐TCMR versus NM‐TCMR, may suggest a synergistic effect of allo‐ and autologous immunity as previously reported [8, 9, 16, 22]. Interestingly, in this cohort enriched with DSA‐positive cases, we did not observe a significant association between DSA MS‐TCMR, possibly due to the underrepresentation of DSA‐negative control cases. However, the presence of non‐HLA antibodies among the DSA‐positive liver transplant recipients may further identify patients with an elevated immunological risk. Our findings confirm the previous reports on kidney [9, 17], liver [8, 13], lung [11], and heart [10] that injuries from antibodies to HLA and non‐HLA antigens may collectively contribute to allograft damage. Our findings that non‐HLA antibodies were elevated in serum paired with MS‐TCMR, but not in mild TCMR, are consistent with previous reports that the mild TCMR may self‐resolve without active treatment, thus it is often less deleterious for long‐term survival. In a large prospective study of 2000 liver allograft biopsies, patients with MS‐TCMR have elevated liver enzyme levels and a higher incidence of subsequent allograft failure compared to those with mild TCMR [23]. Additionally, early MS‐TCMR leads to later TCMR more frequently than mild TCMR [24].

Previously, in a pediatric liver transplant cohort [18], we found that antibodies to SNRPN, GSTT1, and Actin are associated with TCMR. In the current study with an adult liver transplant cohort, anti‐SNRPN and anti‐GSTT1 were found to be associated with MS‐TCMR, but anti‐actin was not. A plausible explanation is that anti‐actin‐associated autoimmune hepatitis often affects children or young adults. Anti‐GSTT1 antibodies have been reported to be associated with central perivenulitis in adult liver transplant biopsies [25]. In the current study, we found biopsies accompanied by positive anti‐GSTT1 antibodies exhibited more frequent mild‐to‐severe central perivenulitis (11/17, 64.7%) than those with negative anti‐GSTT1 antibodies (34/86, 39.5%) (Table S4, *p* = 0.056). Both anti‐GSTT1 and anti‐ARHGDIB could be an allogenic response, as both genes are polymorphic [26, 27, 28]. Notably, anti‐ARHGDIB augmented the risk of renal allograft failure in patients with positive DSA, indicating a synergistic effect [26]. Anti‐α‐enolase antibodies have been reported in classical autoimmune diseases, including rheumatoid arthritis, lupus nephritis, and primary glomerulonephritis [29].

Liver expression of the panel‐18 non‐HLA antigen was summarized in Table S5. Several publications have reported the protein expression of GSTT1 in liver tissue, particularly in hepatocytes [25, 27]. ARHGDIB is involved in the cytoskeleton organization of liver stellate cells [30] and may contribute to liver fibrosis [31]. α‐Enolase is expressed in the liver; its tissue expression and serum antibody to α‐Enolase are both elevated in liver cancers [32]. The other 15 non‐HLA antigens are expressed in liver cholangiocytes and/or hepatocytes, either at the protein level or by single‐cell RNA sequencing based on the Human Protein Atlas (https://www.proteinatlas.org/) (Table S5). These data suggest that non‐HLA antigens expressed in liver tissue may be exposed to the immune system under stress or injury, thereby eliciting humoral immune responses. However, the sequential trends of these antibodies and their temporal relationship with rejection in liver allografts were not assessed in this study. Thus, our observation suggests association rather than causality.

The possible mechanism of non‐HLA antibodies in liver allograft TCMR may involve microvascular inflammation, similar to the previously reported association between non‐HLA antibodies and MVI in renal allografts [9]. Microvascular inflammation has been part of the diagnostic criteria for TCMR of liver allografts since 1997^19^. Recently, there have been ample publications on macrovascular inflammation in antibody‐mediated rejection (AMR) of the kidney allograft, with or without DSA [33]. Hence, it is plausible that DSA and/or non‐HLA antibodies may contribute to microvascular inflammation and, eventually, to AMR or mixed AMR/TCMR in the liver allografts. Future studies, including consistent AMR scoring and C4d testing, would answer these questions.

We acknowledge that the experimental Luminex panel of non‐HLA antibodies requires full validation to demonstrate its analytical and clinical validity, as recommended in Sensitization in Transplantation: Assessment of Risk (STAR) Report [34]. Recently, Obrisca et al. evaluated the two Luminex non‐HLA panels, provided by Immucor and One Lambda, respectively, and their associations with antibody‐mediated rejection in a kidney transplant cohort [35]. They found substantial assay heterogeneity and discrepancies between the two panels, raising concerns about the reproducibility and interpretability of the tests. We have demonstrated an association between non‐HLA antibodies and detrimental outcomes in lung and liver transplant recipients, using either Immucor [11, 13] or One Lambda [18] non‐HLA panels. Only the Immucor Luminex panel was used in the current study. Two of the panel‐18 non‐HLA antibodies, anti‐HARS (Histidyl‐tRNA synthetase) and anti‐SNRPN (Small Nuclear Ribonucleoprotein Polypeptide N), were verified with well‐characterized reference serums tested by standard autoantibody tests (Xu, Q, Shurin, M, Zeevi, A., Unpublished data). In 7 reference serum samples (two were positive for anti‐HARS, three for anti‐SNTPN, and two were negative for both) tested with the Luminex panel, concordance with standard autoantibody tests was 100%. This preliminary work supports the partial analytical validity for at least some of the non‐HLA antibodies tested in the Luminex panel. The clinical validity of the non‐HLA panel may require validating each non‐HLA antibody using antisera with antibody reactivity confirmed by well‐established third‐party methods. A lack of such an antiserum, especially for non‐classical autoantibodies, may necessitate a multidisciplinary collaboration among investigators.

Our cohort was enriched for DSA‐positive for‐cause biopsies, thus lacking control cases with normal biopsies or negative DSA. Abnormal clinical and pathological findings triggered the DSA test; thus, the incidence of positive DSA is very high in this cohort. Due to the high prevalence of DSA, this cohort is underpowered to investigate the impact of non‐HLA antibodies in the absence of DSA. The profile of non‐HLA antibodies and their relationship with DSA in normal adult liver allograft biopsies remains to be further investigated.

In summary, circulating antibodies to non‐HLA antigens, such as AT1R or non‐HLA panels, were associated with an augmented incidence of MS‐TCMR. Liver transplant recipients with positive DSA and non‐HLA antibodies may have normal liver function tests. However, the presence of smoldering active immunity to the liver allograft may exclude these patients as candidates for weaning immunosuppression. If these patients also have elevated liver enzymes, they may deserve fine‐tuning of immunosuppression and probably trigger a liver biopsy to assess the damage in the tissue. We acknowledge that the causal relationship with TCMR remains undetermined; therefore, more evidence is needed to determine whether DSA and non‐HLA antibodies can be used as a biomarker for TCMR risk stratification. Nonetheless, our work highlights the unmet need for large prospective, longitudinal cohort studies that monitor liver function, DSA, non‐HLA antibodies, and biopsy results. Specifically, testing DSA and non‐HLA antibodies in pre‐transplant and sequential posttransplant serum samples would be essential to determine causality and validate the utility of these serological biomarkers in improving posttransplant management.

## Author Contributions

Participated in research design: Qingyong Xu, Nigar A. Khurram, Anthony J. Demetris, Adriana Zeevi. Participated in the writing of the paper: Qingyong Xu, Nigar A. Khurram, Anthony J. Demetris, Adriana Zeevi. Participated in the performance of the research: Carol Bentlejewski, Nigar A. Khurram, Qingyong Xu. Participated in data analysis: Qingyong Xu, Nigar A. Khurram.

## Funding

The authors have nothing to report

## Conflicts of Interest

The authors declare no conflicts of interest relevant to this work.

## Supporting information




**Supporting File 1:** ctr70480‐sup‐0001‐SuppMat.docx

## Data Availability

The data that support the findings of this study are available on request from the corresponding author. The data are not publicly available due to privacy or ethical restrictions.

## References

[1] C. Lefaucheur , K. Louis , A. B. Morris , et al., “Clinical Recommendations for Posttransplant Assessment of Anti‐HLA (Human Leukocyte Antigen) Donor‐Specific Antibodies: A Sensitization in Transplantation: Assessment of Risk Consensus Document,” American Journal of Transplantation 23, no. 1 (2023): 115–132. 36695614 10.1016/j.ajt.2022.11.013

[2] B. D. Tait , C. Susal , H. M. Gebel , et al., “Consensus Guidelines on the Testing and Clinical Management Issues Associated With HLA and Non‐HLA Antibodies in Transplantation,” Transplantation 95, no. 1 (2013): 19–47. 23238534 10.1097/TP.0b013e31827a19cc

[3] T. Taner , M. D. Stegall , and J. K. Heimbach , “Antibody‐Mediated Rejection in Liver Transplantation: Current Controversies and Future Directions,” Liver Transplantation 20, no. 5 (2014): 514–527. 24470340 10.1002/lt.23826

[4] J. G. O'Leary , A. J. Demetris , L. S. Friedman , et al., “The Role of Donor‐Specific HLA Alloantibodies in Liver Transplantation,” American Journal of Transplantation 14, no. 4 (2014): 779–787. 24580828 10.1111/ajt.12667 PMC4412601

[5] A. J. Demetris , “Longterm Outcome of the Liver Graft: The Pathologist's Perspective,” Liver Transplantation 23, no. S1 (2017): S70–S75. 28834080 10.1002/lt.24851

[6] Q. Y. Xu , B. Shrum , S. Leckie , A. Skaro , and V. C. McAlister , “The Impact of Alloantibodies Directed Against the Second Donor on Long‐Term Outcomes of Repeat Liver Transplantation,” Hepatobiliary Surgery and Nutrition 8, no. 3 (2019): 246–252. 31245404 10.21037/hbsn.2019.01.14 PMC6561887

[7] C. O. C. Bellamy , J. G. O'Leary , O. Adeyi , et al., “Banff 2022 Liver Group Meeting Report: Monitoring Long‐Term Allograft Health,” American Journal of Transplantation 24, no. 6 (2024): 905–917. 38461883 10.1016/j.ajt.2024.03.008

[8] J. G. O'Leary , A. J. Demetris , and A. Philippe , “Non‐HLA Antibodies Impact on C4d Staining, Stellate Cell Activation and Fibrosis in Liver Allografts,” Transplantation 101, no. 10 (2017): 2399–2409. 28665894 10.1097/TP.0000000000001853

[9] C. Lefaucheur , D. Viglietti , Y. Bouatou , et al., “Non‐HLA Agonistic Anti‐Angiotensin II Type 1 Receptor Antibodies Induce a Distinctive Phenotype of Antibody‐Mediated Rejection in Kidney Transplant Recipients,” Kidney International 96 (2019): 189–201. 31005275 10.1016/j.kint.2019.01.030

[10] C. L. Butler , M. J. Hickey , N. Jiang , et al., “Discovery of Non‐HLA Antibodies Associated With Cardiac Allograft Rejection and Development and Validation of a Non‐HLA Antigen Multiplex Panel: From Bench to Bedside,” American Journal of Transplantation 20, no. 10 (2020): 2768–2780. 32185871 10.1111/ajt.15863 PMC7494540

[11] Q. Xu , M. Elrefaei , J. L. Taupin , et al., “Chronic Lung Allograft Dysfunction Is Associated With an Increased Number of Non‐HLA Antibodies,” The Journal of Heart and Lung Transplantation 43, no. 4 (2024): 663–672. 38141896 10.1016/j.healun.2023.12.007

[12] D. K. Nayak , P. B. Saravanan , S. Bansal , B. Naziruddin , and T. Mohanakumar , “Autologous and Allogenous Antibodies in Lung and Islet Cell Transplantation,” Frontiers in Immunology 7 (2016): 650. 28066448 10.3389/fimmu.2016.00650 PMC5179571

[13] Q. Xu , V. C. McAlister , A. A. House , M. Molinari , S. Leckie , and A. Zeevi , “Autoantibodies to LG3 are Associated With Poor Long‐Term Survival After Liver Retransplantation,” Clinical transplantation 35, no. 7 (2021): e14318. 33871888 10.1111/ctr.14318

[14] Q. Y. Xu , V. C. McAlister , S. Leckie , A. A. House , A. Skaro , and P. Marotta , “Angiotensin II Type I Receptor Agonistic Autoantibodies are Associated With Poor Allograft Survival in Liver Retransplantation,” American Journal of Transplantation 20, no. 1 (2020): 282–288. 31419065 10.1111/ajt.15571

[15] L. J. Wozniak , M. J. Hickey , A. P. Chan , et al., “Angiotensin II Type‐1 Receptor Antibodies are Associated with Active Allograft Dysfunction Following Pediatric Liver Transplantation,” Transplantation 104, no. 12 (2020): 2547–2556. 32101982 10.1097/TP.0000000000003206

[16] C. Lefaucheur , K. Louis , A. Philippe , A. Loupy , and P. T. Coates , “The Emerging Field of Non‐Human Leukocyte Antigen Antibodies in Transplant Medicine and Beyond,” Kidney International 100, no. 4 (2021): 787–798. 34186057 10.1016/j.kint.2021.04.044

[17] A. Senev , B. Ray , E. Lerut , et al., “The Pre‐Transplant Non‐HLA Antibody Burden Associates With the Development of Histology of Antibody‐Mediated Rejection After Kidney Transplantation,” Frontiers in Immunology 13 (2022): 809059. 35250981 10.3389/fimmu.2022.809059 PMC8888449

[18] Q. Xu , S. M. Bedoyan , C. Bentlejewski , et al., “The Impact of Donor‐Specific Antibody and Non‐HLA Antibodies on Acute Cellular Rejection in Pediatric Liver Transplantation,” Human Immunology 86, no. 3 (2025): 111289. 40157164 10.1016/j.humimm.2025.111289

[19] A. J. Demetris , K. P. Batts , A. P. Dhillon , et al., “Banff Schema for Grading Liver Allograft Rejection: An International Consensus Document,” Hepatology 25, no. 3 (1997): 658–663. 9049215 10.1002/hep.510250328

[20] J. G. O'Leary , H. Kaneku , L. Jennings , B. M. Susskind , P. I. Terasaki , and G. B. Klintmalm , “Donor‐Specific Alloantibodies are Associated With Fibrosis Progression After Liver Transplantation in hepatitis C Virus‐Infected Patients,” Liver Transplantation 20, no. 6 (2014): 655–663. 24678017 10.1002/lt.23854

[21] H. Kaneku , J. G. O'Leary , and N. Banuelos , “De Novo Donor‐Specific HLA Antibodies Decrease Patient and Graft Survival in Liver Transplant Recipients,” American Journal of Transplantation 13, no. 6 (2013): 1541–1548. 23721554 10.1002/ajt.12212 PMC4408873

[22] A. M. Jackson , C. Wiebe , and M. J. Hickey , “The Role of Non‐HLA Antibodies in Solid Organ Transplantation: A Complex Deliberation,” Current Opinion in Organ Transplantation 25, no. 6 (2020): 536–542. 33044346 10.1097/MOT.0000000000000811

[23] A. J. Demetris , K. Ruppert , I. Dvorchik , et al., “Real‐Time Monitoring of Acute Liver‐Allograft Rejection Using the Banff Schema,” Transplantation 74, no. 9 (2002): 1290–1296. 12451268 10.1097/00007890-200211150-00016

[24] C. C. Jadlowiec , P. E. Morgan , A. K. Nehra , et al., “Not all Cellular Rejections are the Same: Differences in Early and Late Hepatic Allograft Rejection,” Liver Transplantation 25, no. 3 (2019): 425–435. 30615251 10.1002/lt.25411

[25] M. Rodriguez‐Mahou , M. Salcedo , E. Fernandez‐Cruz , et al., “Antibodies Against Glutathione S‐Transferase T1 (GSTT1) in Patients With GSTT1 Null Genotype as Prognostic Marker: Long‐Term Follow‐Up after Liver Transplantation,” Transplantation 83, no. 8 (2007): 1126–1129. 17452905 10.1097/01.tp.0000259963.47350.da

[26] A. Senev , H. G. Otten , E. G. Kamburova , et al., “Antibodies Against ARHGDIB and ARHGDIB Gene Expression Associate With Kidney Allograft Outcome,” Transplantation 104, no. 7 (2020): 1462–1471. 31651716 10.1097/TP.0000000000003005

[27] I. Aguilera , J. M. Sousa , and A. Núñez‐Roldán , “Clinical Relevance of GSTT1 Mismatch in Solid Organ and Hematopoietic Stem Cell Transplantation,” Human Immunology 74, no. 11 (2013): 1470–1473. 23792056 10.1016/j.humimm.2013.06.004

[28] E. G. Kamburova , M. L. Gruijters , T. Kardol‐Hoefnagel , et al., “Antibodies Against ARHGDIB are Associated With Long‐Term Kidney Graft Loss,” American Journal of Transplantation 19, no. 12 (2019): 3335–3344. 31194283 10.1111/ajt.15493 PMC6899679

[29] A. Angeletti , P. Migliorini , M. Bruschi , et al., “Anti‐Alpha Enolase Multi‐Antibody Specificity in Human Diseases. Clinical Significance and Molecular Mechanisms,” Autoimmunity Reviews 20, no. 12 (2021): 102977. 34718161 10.1016/j.autrev.2021.102977

[30] M. Kato , H. Iwamoto , N. Higashi , et al., “Role of Rho Small GTP Binding Protein in the Regulation of Actin Cytoskeleton in Hepatic Stellate Cells,” Journal of Hepatology 31, no. 1 (1999): 91–99. 10424288 10.1016/s0168-8278(99)80168-8

[31] T. Utsunomiya , M. Okamoto , S. Wakiyama , et al., “A Specific Gene‐Expression Signature Quantifies the Degree of Hepatic Fibrosis in Patients With Chronic Liver Disease,” World Journal of Gastroenterology 13, no. 3 (2007): 383–390. 17230606 10.3748/wjg.v13.i3.383 PMC4065892

[32] L. Zhang , T. Lu , Y. Yang , and L. Hu , “Alpha‐Enolase Is Highly Expressed in Liver Cancer and Promotes Cancer Cell Invasion and Metastasis,” Oncology Letters 20, no. 5 (2020): 152. 32934720 10.3892/ol.2020.12003 PMC7471668

[33] G. A. Böhmig , A. Loupy , M. Sablik , and M. Naesens , “Microvascular Inflammation in Kidney Allografts: New Directions for Patient Management,” American Journal of Transplantation 25, no. 7 (2025): 1410–1416. 40199388 10.1016/j.ajt.2025.03.031

[34] A. R. Tambur , P. Campbell , A. S. Chong , et al., “Sensitization in Transplantation: Assessment of Risk (STAR) 2019 Working Group Meeting Report,” American Journal of Transplantation 20, no. 10 (2020): 2652–2668. 32342639 10.1111/ajt.15937 PMC7586936

[35] B. Obrișcă , N. Leca , E. Chou‐Wu , et al., “Heterogeneity of Non‐HLA Antibody Prevalence in Kidney Antibody‐Mediated Rejection With the Commercial Luminex Assays,” Transplantation 109, no. 8 (2025): e409–e421. 40128172 10.1097/TP.0000000000005363

