# Supplementary material for: The Presence of Non‐HLA Antibody With DSA Is Associated With Moderate to Severe T Cell‐Mediated Rejection in Liver Transplant Recipients
Source: Clin Transplant. 2026 Feb 15;40(2):e70480. doi: 10.1111/ctr.70480 (PMC12906860; doi:10.1111/ctr.70480)
Supplement: Supplementary file 1 — Supporting File 1: ctr70480‐sup‐0001‐SuppMat.docx [file CTR-40-e70480-s001.docx]

## Supplemental Tables and Figures

Table S1: Distribution of 103 biopsies in 78 patients

| # of biopsies per patient | # of patients | % out of 78 |
| --- | --- | --- |
| 1 | 62 | 79.5 |
| 2 | 9 | 11.5 |
| 3 | 6 | 7.7 |
| 5 | 1 | 1.3 |

Table S2, Demographics of studied patients

|  | n=78 (100%) |
| --- | --- |
| Age, Mean+/-SD | 53.2 +/-13.9 |
| Male Gender, n (%) | 48 (60.8) |
| Re-graft, n (%) | 7 (8.9) |
| Race, n (%) |  |
| White | 68 (86.1) |
| Black | 8 (10.1) |
| Unknown | 3 (3.8) |
| Primary Diagnostics, n (%) |  |
| Virial | 7 (8.9) |
| PSC | 10 (12.7) |
| NASH | 18 (22.8) |
| Alcoholic Cirrhosis | 23 (29.1) |
| Malignancy | 15 (19.0) |
| Others | 6 (7.6) |

NASH: Non-alcoholic steatohepatitis; PSC: Primary Sclerosing Cholangitis.

Others: Autoimmune (n=2), Primary Biliary Cirrhosis (PBC, n=1), Cryptogenic (n=2), Wilson's Disease (n=1).

Figure S1A. Anti-AT1R antibodies were elevated in Moderate-Severe TCMR (T cell-mediated rejection). *P <* 0.01, Mann-Whitney Test. AT1R, Angiotensin II type 1 receptor.


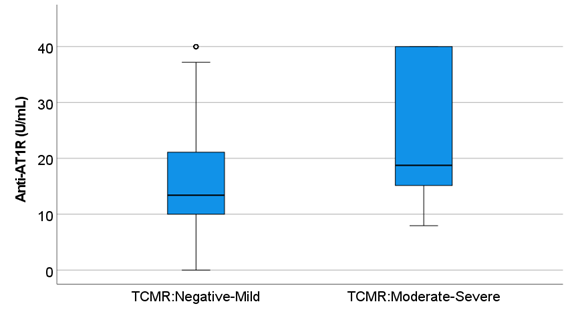


Figure S1B. Patients with Moderate-to-Severe TCMR had an increased number of Panel-18 non-HLA Antibodies. *P* < 0.001, Mann-Whitney Test


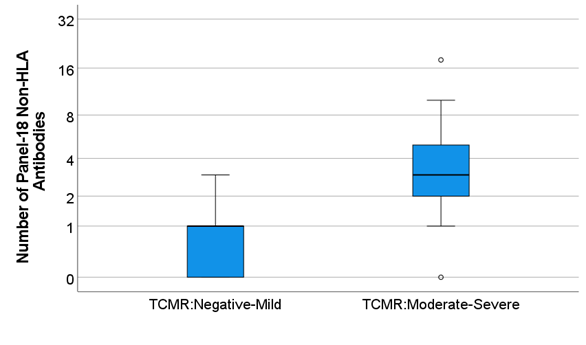


Table S3: DSA is not significantly associated with MS-TCMR

|  | NM-TCMR (n=77) | MS-TCMR (n=26) | HR (95% CI) | *p* |
| --- | --- | --- | --- | --- |
| DSA MFI > 1 K |  |  |  |  |
| Negative | 5 | 3 | 1 |  |
| Positive | 72 | 23 | 0.53 (0.12 – 2.40) | 0.412 |
| DSA MFI > 5 K |  |  |  |  |
| Negative | 31 | 8 | 1 |  |
| Positive | 46 | 18 | 1.28 (0.80 – 2.06) | 0.302 |
| DSA MFI > 10 K |  |  |  |  |
| Negative | 47 | 16 | 1 |  |
| Positive | 30 | 10 | 0.98 (0.39 – 2.44) | 0.964 |
| C1Q DSA |  |  |  |  |
| Negative | 51 | 14 | 1 |  |
| Positive | 26 | 12 | 1.68 (0.68 – 4.15) | 0.26 |

HR (hazard ratio), 95% CI (Confidence Interval), and *p*-values were calculated using binomial logistic regression.

DSA, donor-specific antibody, only DSA in the biopsy-paired serum was analyzed for its association with TCMR; NM-TCMR, negative-mild T cell-mediated rejection; MFI, mean fluorescence intensity; MS-TCMR, moderate-severe T cell-mediated rejection.

Table S4

|  | Anti-GSTT1 Antibody | |  |
| --- | --- | --- | --- |
|  | Negative (n=86) | Positive (n=17) | *p*-val |
| Central Perivenulitis |  |  | 0.056 |
| None/minimal | 52 | 6 |  |
| Mild/moderate/severe | 34 | 11 |  |

*P-values* were calculated using a two-sided Chi-square test.

GSTT1, Glutathione S-Transferase theta-1;

Table S5: Liver tissue expression of non-HLA antigen targets

| Antigen | Antigen Detail | Protein Level | Single-cell RNA |
| --- | --- | --- | --- |
| AGRIN | Agrin | Cholangiocytes (Low); Hepatocytes (Medium) | Cholangiocytes, Hepatocytes, Kupffer cells |
| ARHGDIB | Rho GDP dissociation inhibitor beta | Stellate cells (Ref. #27, 28) | Cholangiocytes, Hepatocytes, Kupffer cells |
| ATP5B | ATP synthase, beta polypeptide | Cholangiocytes (high); Hepatocytes (high) | Cholangiocytes, Hepatocytes, Kupffer cells |
| CD40 | CD40 molecule, TNF receptor superfamily member 5 | Cholangiocytes (ND); Hepatocytes (ND) | Cholangiocytes, Hepatocytes, Kupffer cells |
| CXCL9 | C-X-C Motif Chemokine 9 | NA | Hepatocytes, Kupffer cells |
| DEXI | Dexamethasone-induced transcript | Cholangiocytes (Medium); Hepatocytes (Medium) | Cholangiocytes, Hepatocytes, Kupffer cells |
| ENO1 | Alpha-enolase | Cholangiocytes (Medium); Hepatocytes (Medium); Ref. #29 | Cholangiocytes, Hepatocytes, Kupffer cells |
| FLRT2 | Leucine-rich repeat transmembrane protein FLRT2 | NA | Cholangiocytes, Kupffer cells |
| GSTT1 | Glutathione S-Transferase theta-1 | Hepatocytes (Ref. # 22, 24) | NA |
| HARS | Jo-1 | Cholangiocytes (Medium); Hepatocytes (Medium) | Cholangiocytes, Hepatocytes, Kupffer cells |
| LGALS3 | Lectin, galactoside-binding, soluble, 3 | Cholangiocytes (Low); Hepatocytes (ND) | Cholangiocytes, Hepatocytes, Kupffer cells |
| NCL | Nucleolin | Cholangiocytes (high); Hepatocytes (high) | Cholangiocytes, Hepatocytes, Kupffer cells |
| P2RY11 | Purinergic receptor P2Y, G-protein coupled, 11 | Cholangiocytes (ND); Hepatocytes (ND) | Cholangiocytes, Hepatocytes, Kupffer cells |
| PLA2R1 | Phospholipase A2 receptor 1, 180kDa | Cholangiocytes (ND); Hepatocytes (ND) | Cholangiocytes |
| PTPRO | Receptor-type Tyrosine-protein Phosphatase U | Cholangiocytes (ND); Hepatocytes (ND) | Cholangiocytes, Kupffer cells |
| SHC3 | SHC Adaptor Protein 3 | Cholangiocytes (ND); Hepatocytes (Medium) | ND |
| SNRPN | Small Nuclear Ribonucleoprotein Polypeptide N (Smith antigen core) | Cholangiocytes (ND); Hepatocytes (ND) | Cholangiocytes, Hepatocytes |
| STAT6 | Signal Transducer and Activator of Transcription 6 | Cholangiocytes (ND); Hepatocytes (ND) | Cholangiocytes, Hepatocytes, Kupffer cells |

All expression data were based on the Human Protein Atlas (https://www.proteinatlas.org/), unless specifically noted with a reference (Ref.#).

NA, Data is not available in Human Protein Atlas; ND, not detected.
